# Supplementary material for: Age-Related Glucose Intolerance Is Associated with Impaired Insulin Secretion in Community-Dwelling Japanese Adults: The Kumamoto Koshi Study
Source: Biomedicines. 2025 Feb 6;13(2):380. doi: 10.3390/biomedicines13020380 (PMC11852980; doi:10.3390/biomedicines13020380)
Supplement: Supplementary file 1 [file biomedicines-13-00380-s001.zip › biomedicines-3445774-supplementary.pdf]

biomedicines

MDPI

Supplementary Materials

Supplementary Table S1. Multiple regression analysis for indices of insulin resistance

	HOMA-IR		Matsuda Index	
	**Std β**	***p***	**Std β**	***p***
Age	−0.023	0.140	0.011	0.636
Sex	0.013	0.829	0.023	0.800
BMI	0.091	**0.002**	−0.122	**0.005**
Percent Body Fat	−0.002	0.935	−0.001	0.976
Skeletal Muscle Index	−0.064	0.116	0.100	0.109
Calf Circumference	0.010	0.745	−0.009	0.837
Grip Strength	0.022	0.383	−0.054	0.168

The standardized regression coefficient (Std β) and each probability value (*p*) are shown. Statistically significant results ($p < 0.05$) are shown in bold. Individuals with NGT (n = 106) are included in the analysis. Body composition data were available for 63 NGT individuals.
